# Multi-Resonant Full-Solar-Spectrum Perfect Metamaterial Absorber

**DOI:** 10.3390/nano14231959

**Published:** 2024-12-06

**Authors:** Zhe Shen, Junfan Ni

**Affiliations:** School of Electronic and Optical Engineering, Nanjing University of Science and Technology, Nanjing 210094, China; nijunfan@njust.edu.cn

**Keywords:** multi-resonance, perfect absorption absorber, metamaterial

## Abstract

Currently, perfect absorption properties of metamaterials have attracted widespread interest in the area of solar energy. Ultra-broadband absorption, incidence angle insensitivity, and polarization independence are key performance indicators in the design of the absorbers. In this work, we proposed a metamaterial absorber based on the absorption mechanism with multiple resonances, including propagation surface plasmon resonance (PSPR), localized surface plasmon resonance (LSPR), electric dipole resonance (EDR), and magnetic dipole resonance (MDR). The absorber, consisting of composite nanocylinders and a microcavity, can perform solar energy full-spectrum absorption. The proposed absorber obtained high absorption (>95%) from 272 nm to 2742 nm at normal incidence. The weighted absorption rate of the absorber at air mass 1.5 direct in the wavelength range of 280 nm to 3000 nm exceeds 98.5%. The ultra-broadband perfect absorption can be ascribed to the interaction of those resonances. The photothermal conversion efficiency of the absorber reaches 85.3% at 375 K. By analyzing the influence of the structural parameters on the absorption efficiency, the absorber exhibits excellent fault tolerance. In addition, the designed absorber is insensitive to polarization and variation in ambient refractive index and has an absorption rate of more than 80% at the incident angle of 50°. Our proposed absorber has great application potential in solar energy collection, photothermal conversion, and other related areas.

## 1. Introduction

Solar energy is widely considered an energy source on which humans rely due to its relative renewability and minimal environmental repercussions [1,2,3]. Up to now, the conversion of solar energy into other forms of energy has been greatly developed in applications such as solar cells [4], solar thermophotovoltaic systems [5], solar steam generation [6], and photo-catalytic reactions [7,8]. For solar thermal applications, one of the important technologies is the conversion of light into heat by using a perfect solar absorber [5]. An efficient solar absorber will significantly improve the efficiency of photothermal conversion. According to air mass 1.5 direct (AM 1.5D), i.e., direct radiation from the sun that reaches the surface of the earth, the solar energy is concentrated in the range of 280 nm to 2500 nm. This implies that for efficient absorption of solar energy, the absorber needs to have a high absorption rate within a broad bandwidth from shortwave to longwave. However, the absorption response of conventional solar thermal absorbers [9], such as those using metal particles, multi-layered coatings, and cermets, mostly relies on their intrinsic absorption properties. Their narrow resonances restrict the bandwidths, and the scattering and loss of the structures or configurations affect the high absorption.

In recent years, the rise of metamaterials has brought new opportunities for broadband absorbers [10,11,12]. Metamaterials are sub-wavelength periodic or aperiodic nanostructures designed and fabricated artificially, and they can manipulate the amplitude of specific bands of electromagnetic waves [13,14,15]. In 2008, Landy et al. pioneered the use of metamaterial absorbers with gold open-ring structures for electromagnetic absorption [16], which has a wide range of applications such as biosensing, light detection, and imaging device manufacturing. This single structure resonance produces perfect absorption in the narrow GHz band. Researchers have further proposed using the combination of multiple resonances to obtain broad spectrum absorption in the solar band in the recent decade. Serkan et al. proposed the use of an Ag-SiO_2_-Ag three-layer structure to excite propagation surface plasmon resonance (PSPR) and localized surface plasmon resonance (LSPR), which has an absorption rate of over 90% in the visible (VIS) band [17]. Lei et al. achieved perfect absorption at the rate of 90% at the wavelength band ranging from 354 nm to 1066 nm by combining the PSPR, LSPR, and Fabry–Perot (F–P) resonance [18]. In addition, Feng et al. designed an absorber consisting of dielectric ribbons to excite electric dipole resonance (EDR), Mie resonance, and SPR [19]. This absorber has an average absorption of 97.75% from 382 nm to 1100 nm. The high absorption (>90%) bandwidths of the above metamaterial absorbers are usually below 1000 nm. In order to further broaden the absorption bandwidth, researchers combined a few wideband configurations to obtain the incorporation of more resonances. Liu et al. proposed a metamaterial absorber composed of triple-layer resonators [20]. This absorber obtains more than 90% absorption from 280 nm to 1970 nm by introducing multiple LSPRs excited by resonators with different sizes. Yu et al. designed an absorber comprising multiple circle and rectangle dishes, which has absorption rates above 90% across a wavelength range of 820 nm to 2520 nm [21]. Although all of the above-mentioned works obtained high absorption rates in broadbands, the absorption bandwidths are slightly away from the solar full-spectrum range.

In this paper, we designed a plasmonic multi-resonant metamaterial absorber. The design adopts the most reported structure resonances, including LSPR, PSPR, EDR, and MDR, to achieve ultimate broadband absorption. The absorber consists of composite nanocylinders and a microcavity structure. In this work, we studied the absorption response and thermal emission performance of the absorber, investigated the perfect absorption mechanism, tested the influence of structural parameters, ambient refractive index, and incident angle on the absorption performance, and verified the polarization independence. The designed ultra-broadband metamaterial absorber has conceivable applications in solar thermoelectric generators, solar steam generators, thermal emitters, and so on.

## 2. Materials and Methods

**Figure 1:**
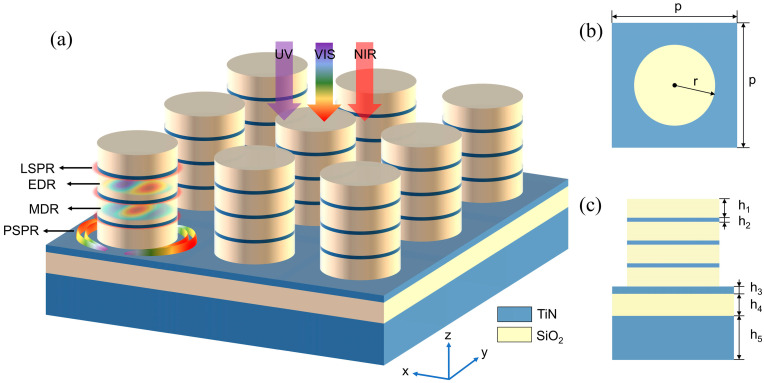
(**a**) Schematic diagram of the proposed metamaterial absorber. Side (**b**) and top (**c**) view of the absorber unit.

The stereoscopic structure of the designed absorber is shown in Figure 1a. The metamaterial structure consists of periodic composite nanocylinders supported by a microcavity. As shown in Figure 1b, the period of the nanocylinders is 500 nm (p) and the radius of the nanocylinder is 200 nm (r). The nanocylinder metamaterial consists of four layers of 70 nm (h_1_) SiO_2_ nanodisks and three layers of 10 nm (h_2_) TiN nanodisks alternately superimposed. The thickness of the three layers in the lower microcavity structure (TiN-SiO_2_-TiN) is 20 nm (h_3_), 60 nm (h_4_), and 200 nm (h_5_), respectively. Under sunlight incidence, the EDR, MDR, PSPR, and LSPR will be excited at different bands of the solar spectrum, resulting in sunlight being confined and localized in the metamaterial absorber. We used the three-dimensional finite-difference time-domain method for simulating the absorber. In the setup of the simulation, the periodic boundary conditions are applied for the lateral boundaries, and the perfectly matched layers are adopted at the top and bottom. The absorber is irradiated by plane waves polarized along the *x*-axis. The mesh step in the simulation is 5 nm, and the dielectric constants of TiN and SiO_2_ are adopted from the experimental data by Palik [22]. TiN has the advantages of being cost effective, having a high melting point, having thermal stability, and having excellent optical absorption properties. SiO_2_ has the advantages of thermal stability and excellent optical transmission properties. The absorptivity (A) of an absorber can be calculated by the formula A = 1 − T − R, where T and R denote the transmissivity and reflectivity, respectively. In the simulation, the TiN substrate is thick enough to prevent the incident light from penetrating the absorber. So, the absorption formula can be shortened to A = 1 − R.

## 3. Results and Discussions

### 3.1. Absorption Response

**Figure 2:**
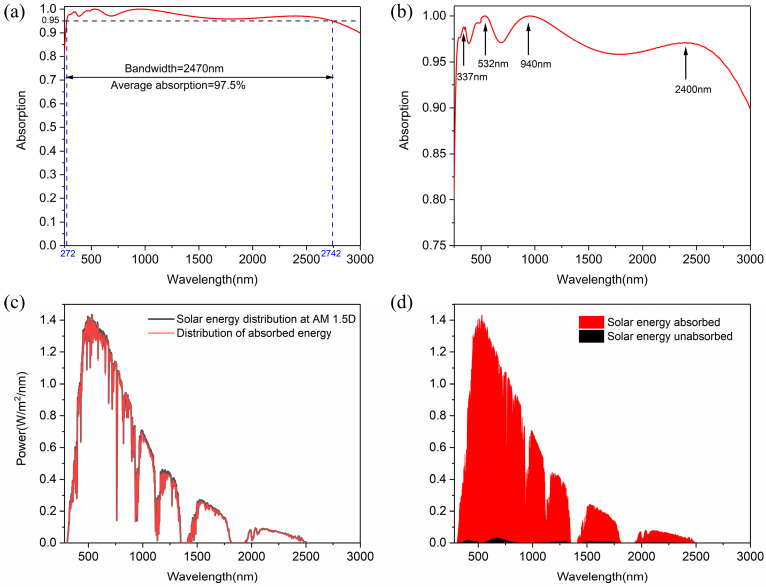
(**a**) The absorption spectrum of the absorber under normal incidence. (**b**) The absorption spectrum on the scale of absorption rate from 0.75 to 1. (**c**) The distribution of solar energy and energy absorbed by the absorber at AM 1.5D. (**d**) Solar energy is absorbed and unabsorbed by the absorber at AM 1.5D.

The absorption response of the designed metamaterial absorber in the wavelength range of 250 nm to 3000 nm is shown in Figure 2a. It can be seen that the overall absorptivity of the absorber in this wavelength range is close to 1. Considering the absorption efficiency of over 95%, the absorption spectrum bandwidth can reach 2470 nm (from 272 nm to 2742 nm). Moreover, the average absorption efficiency of the absorber from 272 nm to 2742 nm reaches 97.42%. Figure 2b shows the absorption spectrum on the scale of absorption rate from 0.75 to 1. There are four main absorption peaks in the absorption spectrum curve of the absorber, and their wavelengths are 338 nm, 532 nm, 940 nm, and 2400 nm, respectively.

In order to test the performance of the absorber under solar radiation, we calculated the absorption spectrum at AM 1.5D. The absorption equation at AM 1.5D is:

(1) $$\eta_{A}=\frac{\int_{\lambda \min}^{\lambda \max}(1-R(\omega ))\cdot I_{\mathrm{AM}1.5D}(\omega )d\omega }{\int_{\lambda \min}^{\lambda \max}I_{\mathrm{AM}1.5D}(\omega )d\omega }.$$

In this equation, *η_A_* denotes the weighted absorption rate, *R*(*ω*) denotes reflectivity, and *I*_AM1.5D_(*ω*) denotes the intensity of sunlight at AM 1.5D; *λ*min and *λ*max denote the minimum and maximum wavelengths, respectively. In the equation, the *λ*max and *λ*min are 280 nm and 3000 nm, respectively. Through calculation, the weighted absorption rate in the range of 280 nm to 3000 nm is over 98%. Figure 2c shows the distribution of solar energy and energy absorbed by the absorber at AM 1.5D. The solar spectrum covers the UV, VIS, and NIR bands, and the main energy is distributed within the range of 280 nm and 2500 nm. Interestingly, the distribution of energy absorbed by the absorber almost coincides with the solar spectrum. Figure 2d clearly shows the solar energy absorbed and unabsorbed by the absorber at AM 1.5D. The tiny unabsorbed energy is mainly distributed in the VIS band, and it may be negligible compared to the whole energy. These results show that the working band of the absorber is compatible with the solar spectrum at AM 1.5D. Therefore, the designed absorber can be applied in solar thermal systems for photothermal conversion.

### 3.2. Thermal Emission and Photothermal Conversion

For solar thermophotovoltaic systems, effective photothermal conversion requires the absorber to possess strong solar absorption and weak thermal emission at the working temperature [9]. Therefore, we investigated the thermal radiation performance of the absorber from 2.5 µm to 20 µm. Assuming the absorber is illuminated by unfocused sunlight and ignoring the convection and heat conduction losses, the thermal emissivity (*ε*) and photo-thermal conversion efficiency (*η*) are as follows [23,24]:

(2) $$\varepsilon =\frac{\int_{2.5\mu m}^{20\mu m}I_{B}(\lambda ,T)A(\lambda )d\lambda }{\int_{2.5\mu m}^{20\mu m}I_{B}(\lambda ,T)d\lambda },$$

(3) $$I_{B}(\lambda ,T)=\frac{2hc^{2}}{\lambda^{5}\left(e^{\frac{hc}{k_{B}T}}-1\right)},$$

(4) $$\eta =\eta_{A}-\varepsilon \frac{\sigma \left(T_{abs}^{4}-T_{sky}^{4}\right)}{CI_{s}},$$

where *I*_B_(*λ*,*T*) denotes radiation intensity of the ideal blackbody, *A*(*λ*) denotes absorptivity, *λ* denotes wavelength of light, *T* denotes absolute temperature, h denotes Planck’s constant, c denotes velocity of light in vacuum, k_B_ denotes Boltzmann constant, σ denotes Stefan–Boltzmann constant, and *C* denotes solar concentration ratio. *I_s_* denotes the total solar irradiance, which is typically valued at 1 kW/m^2^ [25]. *T*_sky_ and *T*_abs_ denote the temperatures of the environment and the absorber, respectively.

**Figure 3:**
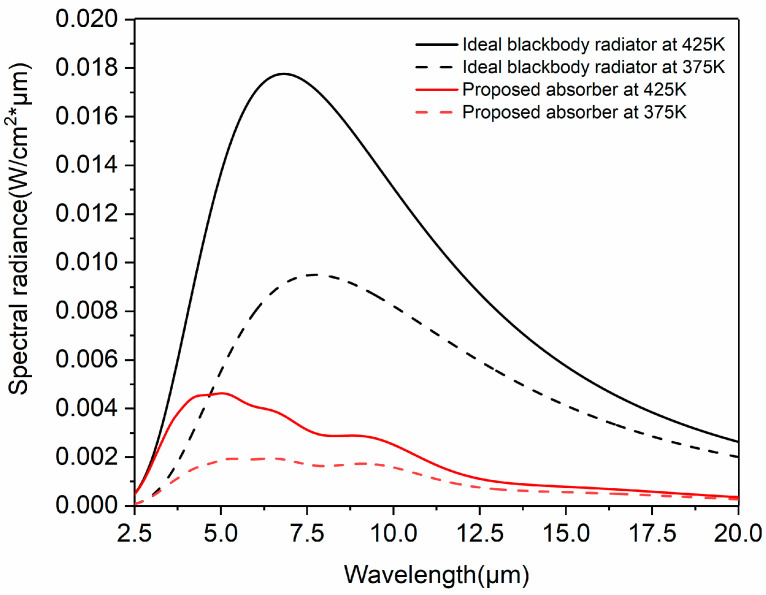
Thermal emission spectrum of the absorber (red) and the ideal blackbody (black) from 2.5 µm to 20 µm at 375 K (dotted line) and 425 K (solid line).

Figure 3 shows the thermal emission spectra of the absorber and the ideal blackbody at 375 K and 425 K from 2.5 µm to 20 µm. The total emittance (*ε*) of the proposed absorber at 425 K and 375 K was calculated according to Equations (2) and (3) to be 19.5% and 21.6%, respectively. Assuming that *C* = 1 and *T*_sky_ = 300 K, the photothermal conversion efficiency (*η*) of the absorber at 425 K and 375 K was calculated according to Equation (4) to be 70.7% and 85.3%, respectively. This result indicates that the absorber has good performance in photothermal conversion. On the other hand, as the temperature increases, the thermal conversion efficiency decreases. However, the absorber still maintains an acceptable thermal conversion performance at a temperature increase of 50 K. This result indicates that the absorber can operate over a range of temperatures.

### 3.3. Physical Mechanism

**Figure 4:**
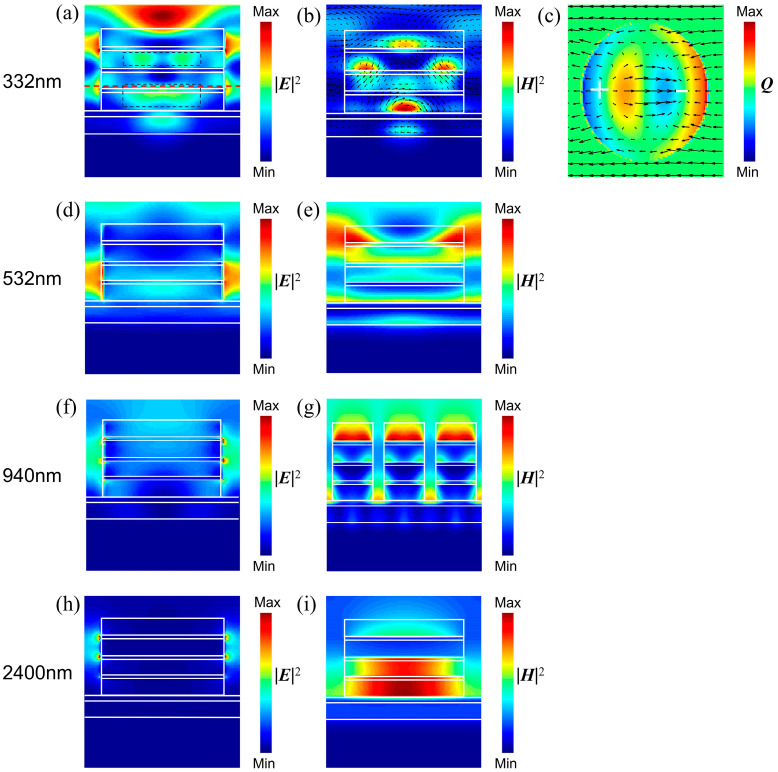
Electric field (**a**,**d**,**f**,**h**) and magnetic field (**b**,**e**,**g**,**i**) distributions of the metamaterial absorber in the x-z plane at 332 nm, 532 nm, 940 nm, and 2400 nm, respectively. (**c**) Charge and electrical field line distributions in the x-y plane at the red line in (**a**). The green color framed by the black dotted lines in (**a**) represents two pairs of local electric fields.

To reveal the mechanism of ultra-broadband absorption, we calculated the distributions of the electric fields and magnetic fields of the absorber at the corresponding wavelengths of the four absorption peaks. As can be seen from Figure 4a, the electric field at 332 nm is mainly distributed between the composite nanocylinder arrays and inside the SiO_2_ nanodisks. Interestingly, there are multiple pairs of local electric fields inside the nanodisks. In Figure 4c, we calculated the distribution of charge and electrical field lines in the x-y plane at the red line in Figure 4a. There is a pair of opposite charges, and the electrical field lines are pointed from the positive charge to the negative charge. This can be regarded as an electric dipole, suggesting the existence of EDR in the UV band. Figure 4b shows the distributions of magnetic field and electrical field lines at 332 nm. The magnetic field is mainly distributed inside the SiO_2_ nanodisks, and there are multiple local fields. Furthermore, the local magnetic field is accompanied by circulating electric field lines. This phenomenon indicates that MDR is excited within the nanodisks. Therefore, the perfect absorption at 332 nm originated from the influence of both EDR and MDR. At 532 nm, the electromagnetic field is mainly distributed between the cylindrical arrays, as shown in Figure 4d,e. This may be explained by the intrinsic absorption of the material. At 940 nm, the electric field is confined to the edges of the TiN nanodisks, as shown in Figure 4f. The energy of the light is localized at the edge of the nanodisks, which is a typical phenomenon of LSPR. In Figure 4g, we calculated the magnetic field distribution in the x-z plane for multiple absorber units. The magnetic field not only presented on the top TiN nanodisk but also propagated on the interface between the cylindrical structure and microcavity, suggesting that PSPR is excited on the TiN film. The LSPR and PSPR correspond to the absorption peak at 940 nm in Figure 2d. For 2400 nm, the magnetic field is localized inside the composite nanocylinder, and the electric field is confined to the edges of the TiN nanodisks in the top and second layers, as shown in Figure 4h,i. The LSPR also corresponds to the absorption peak at 2400 nm in Figure 2d. To sum up, the absorption peaks in Figure 2d are associated with EDR, MDR, PSPR, and LSPR. The ultra-wideband high absorption of the absorber is attributed to the effect of those resonances.

**Figure 5:**
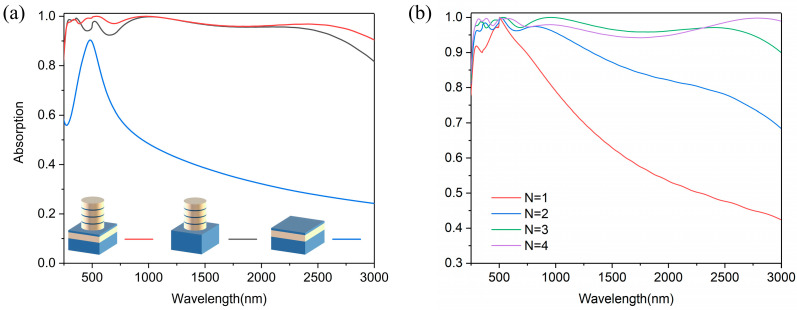
(**a**) Absorption spectrum of the absorber (red), and the absorber without the SiO_2_ layer (gray), and without composite nanocylinder (blue). (**b**) The absorption spectrum of the absorbers with the number of TiN nanodisk layers from 1 to 4.

**Figure 6:**
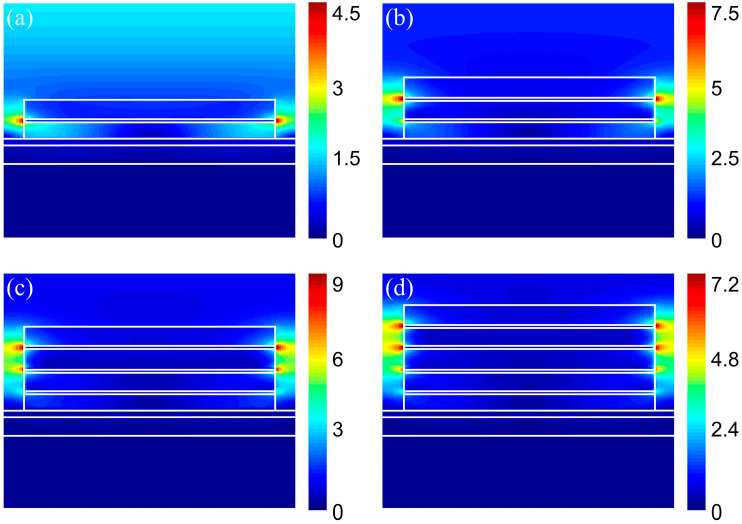
(**a**–**d**) Electric field distributions of the metamaterial absorbers with numbers of TiN nanodisk layers from 1 to 4 in the x-z plane at 2400 nm.

In order to further illustrate the mechanism of ultra-broadband absorption of the absorber, the main components of the absorber, microcavity and composite nanocylinder, are studied. Keeping the rest parameters unchanged, we calculated the absorption spectrum of the absorber by removing the microcavity or composite nanocylinder, as shown in Figure 5a. The absorption spectrum of the microcavity has an absorption peak around 600 nm. Compared to the absorption spectrum of the absorber removing the microcavity, our absorber shows an increase in the absorption rate from 500 nm to 1000 nm and 2000 nm to 2500 nm. Therefore, the microcavity is important for the absorption performance. We also calculated the absorption response of the absorber for the number of TiN nanodisk layers from 1 to 4, as shown in Figure 5b. The absorptivity in the NIR and UV bands is increased with the increasing number of layers. As the number of layers is increased to 3, the absorptivity of the absorber in the solar band is over 95%. When the number of layers is increased to 4, the absorptivity of the absorber within the solar band becomes stable. In order to explain this phenomenon, we also calculated the electric fields of the x-z plane with the number of layers from 1 to 4 at 2400 nm, as shown in Figure 6a–d. It can be seen that the strength of the electric field enhances gradually as the number of layers increases, which is consistent with the result in Figure 5b. The strength of the electric field reaches a maximum at three layers of nanodisk. Thus, three layers of nanodisk were selected for the design of the composite nanocylinder. The microcavity and composite nanocylinder with three layers of nanodisk can ensure excellent absorption performance of the absorber.

In order to compare the absorption performance with those of other recently reported solar absorbers, we list the physical mechanism, high absorption bandwidth, average absorption, and weighted absorption of some previous similar works in Table 1. Previous works adopted a single array structure by incorporating limited resonances. We proposed the metamaterial absorber consisting of a microcavity and composite nanocylinders, adopting the full resonance, including EDR, MDR, LSPR, and PSPR. Considering the absorptivity is greater than 95%, the absorption bandwidth of the absorber is approaching 2500 nm, covering the VIS and NIR bands. Moreover, the weighted absorption rate of the absorber at AM 1.5D reaches up to 98.5%, which is close to perfect absorption across the full-solar spectrum. To sum up, the absorber presents a wide working bandwidth and high absorption efficiency; thus, our proposed absorber has excellent solar spectrum absorption performance.

### 3.4. Influence of Structural Parameters on Absorption Response

**Figure 7:**
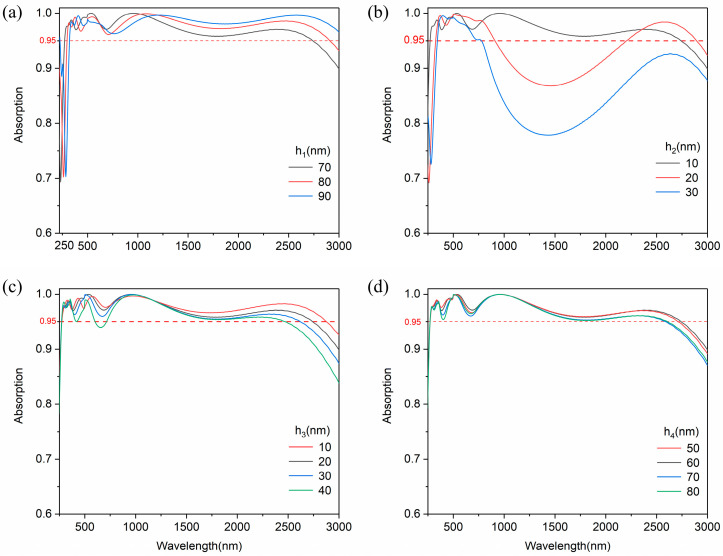
(**a**–**d**) are absorption spectrum under the four cases of different thicknesses of SiO_2_ nanodisk (h_1_), TiN nanodisk (h_2_), TiN film (h_3_), and SiO_2_ film (h_4_).

In the actual fabrication process, manufacturing errors are inevitable. Therefore, we also investigated the influence of structural parameter variation on the absorber performance. As shown in Figure 7a, the absorption spectrum of the absorber shifts with the increasing thickness (h_1_) of SiO_2_ nanodisks, and there is no significant decrease in the absorption in the solar band. From Figure 7b, as the thickness (h_2_) of TiN nanodisks increases to 20 nm and 30 nm, the absorption performance from 800 nm to 2500 nm decreases significantly. This is due to the strength of the LSPR varying with the thickness of the TiN nanodisk, and the thin nanodisk may produce efficient plasmon resonance. From Figure 7c, the variation in TiN film thickness (h_3_) slightly influences the absorption rate in the VIS and mid-infrared bands. TiN film restricts the entry of electromagnetic waves into the SiO_2_ microcavity with the increase in thickness, thus weakening the cavity resonance. In Figure 7d, the absorption response of the absorber remains stable as the thickness (h_4_) of the microcavity varies. These results indicate that our proposed absorber has an excellent fabrication error tolerance for most structural parameters.

**Figure 8:**
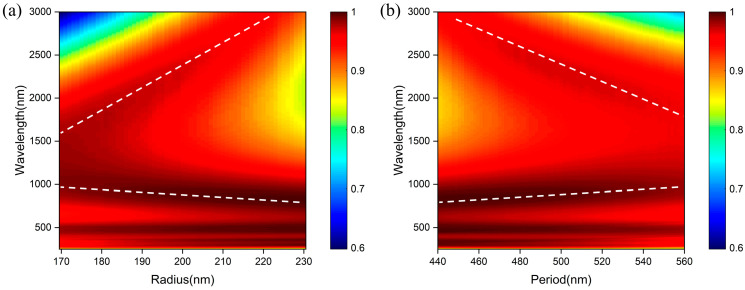
Absorption spectrum map of the absorbers at different structure periods (**a**) and nanocylinder radius (**b**). The write dotted lines donate the resonance wavelengths.

For further detailed analysis, we investigated the influence of structural period and nanocylinder radius on the absorption performance of the proposed absorber. It can be seen from Figure 8a that as the radius of the nanocylinder increases, the absorption peaks around 1000 nm and 2000 nm are blue-shifted and red-shifted, respectively. In Section 3.4, we get that the high absorption around 1000 nm and 2000 nm is attributed to the influences of PSPR and LSPR, respectively. The variation in nanocylinder radius leads to the changing of effective dielectric constant, affecting the excitation wavelength of PSPR and resulting in the variation in the spectrum map around 1000 nm. Meanwhile, the variation in radius also changes the metal structure parameters, affecting the excitation wavelength of LSPR and resulting in the changing of the spectrum map around 2000 nm. From Figure 8b, the changing of the resonance wavelengths due to PSPR or LSPR with the changing of radius and period are reversed. This can be comprehended that the increase in radius is equivalent to the decrease in period, and vice versa. The resonance peaks of PSPR are red-shifted with the increase in period, while those of LSPR are blue-shifted with the increase in period. These results indicate that we can adjust the bandwidth of the absorption spectrum to meet the requirements of various operating wavelengths by changing the nanocylinder radius and structural period.

### 3.5. Polarization-Independent and Angle-Insensitive

**Figure 9:**
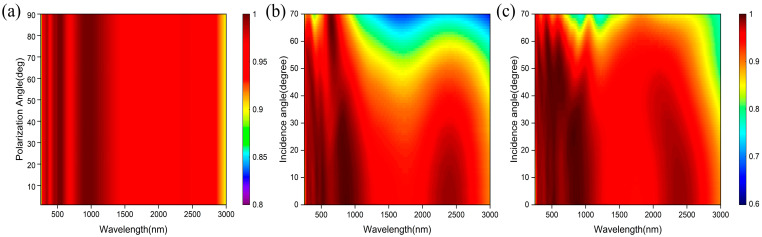
(**a**) The absorption spectrum map of the absorber at different polarization angles (0–90°) of incidence. Absorption spectrum map of the absorber to different angles (0–70°) of incidence in TM mode (**b**) and TE mode (**c**).

In practical applications, the polarization direction and incident angle change over time. As shown in Figure 9a, we calculated the absorption spectrum map for polarization angles from 0° (TM-polarized) to 90° (TE-polarized) at normal incidence. It can be seen that the absorption response of the absorber remains stable with the different angles of polarization from 250 nm to 3000 nm. The identical response of the absorber to different polarized light is derived from the symmetry of the structure [30]. This result shows the absorber is unaffected by polarization. Figure 9b,c shows the absorption spectrum map of oblique incidence from 0° to 70° under TM and TE modes. For TM and TE modes, the absorption response of the absorber can remain relatively stable at incident angles from 0° to 40°, indicating the insensitivity of the absorber to small angles. When the incident angle is increased to 50°, the absorption rate of the absorber in the wavelength range of 280 nm to 2500 nm is still greater than 80%, maintaining a good absorption performance to a large incident angle. Moreover, the absorption band is blue-shifted as the angle of incidence increases, which is consistent with the phenomenon reported in previous work [19]. The reason is that as the angle of incidence increases, the effective dielectric constant of the waveguide layer decreases. To conclude, our absorber has the advantages of being polarization independent, relatively incidence angle insensitive, and operating properly at a large incident angle.

### 3.6. Influence of Ambient Refractive Index on Absorption Response

**Figure 10:**
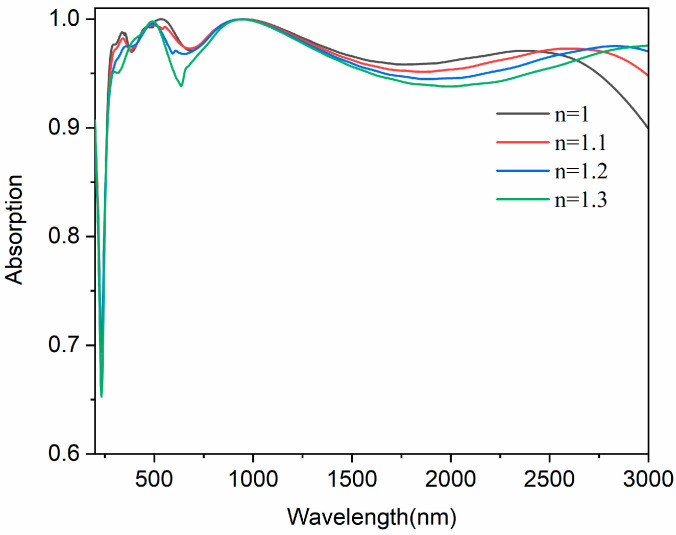
The absorption spectrum of the absorber at ambient refractive indexes from 1 to 1.3.

In practical applications, different weather conditions can cause the variation in environmental refractive index, affecting the absorption performance of the absorber. Therefore, we investigated the influence of ambient refractive index on the absorber. Figure 10 shows the absorption spectrum of the designed absorber at ambient refractive index from 1 to 1.3. The absorption spectrum of the absorber in the main band changes slightly as the refractive index increases. The absorption spectrum in mid-infrared is shifted as the refractive index changes, but the absorber remains consistent in absorption spectrum, maintaining a high absorption rate (>90%) in the solar band. This result indicates that the designed absorber is comparatively insensitive to environmental changes and can be applied in a wide range of backgrounds.

## 4. Conclusions

In conclusion, we designed a multi-resonant, ultra-broadband metamaterial absorber for full-solar-spectrum absorption. The absorber consists of composite nanocylinders supported by a microcavity structure. Our proposed absorber exhibits absorption rates exceeding 95% in the wavelength range of 272 nm to 2742 nm, obtaining high absorption that effectively covers the solar spectrum. The weighted absorption of the absorber in the range of sunlight energy is as high as 98.5%. Moreover, the absorber has a total thermal emissivity of 21.6% and a photothermal conversion efficiency of 85.3%. By analyzing the electromagnetic field distribution of the absorption peaks, we found that EDR and MDR correspond to absorption peak in the UV band, and PSPR and LSPR correspond to absorption peaks in the NIR band. Attributed to multiple resonances, the absorber obtains perfect absorption of the full solar spectrum. In addition, the absorber exhibits excellent error tolerance for the structural parameters. Moreover, our proposed absorber is polarization independent, incidence angle insensitive, and maintains a good absorption response at a large incident angle. Additionally, the absorber is also insensitive to the variation in ambient refractive index. The proposed absorber has conceivable applications in solar photothermal conversion.

## Figures and Tables

**Table 1:** Performance comparison of different metamaterial solar absorbers.

Structures	Physical Mechanism	Range (Absorption > 90%)	Range (Absorption > 95%)	Absorption at AM 1.5
Tungsten nanosphere [26]	LSPR, F-PR, gap SPR	1747 nm	1570 nm	/
Four-Corner Star Array [27]	LSPR, F-PR, plasma near-field coupling	1600 nm	1391 nm	97.3%
Three layers ring [28]	/	2452 nm	/	98.96%
Disk and cube structure [21]	LSPR, PSPR	1759 nm	/	97.60%
Elliptic array [29]	/	1650 nm	/	88.16%
Composite nanocylinder and microcavity structure	LSPR, PSPR, EDR, MDR	2740 nm	2465 nm	98.5%

## Data Availability

The data are available on reasonable request from the corresponding author.
